# Scope and Limitations of Cerebral Surgery

**Published:** 1893-09

**Authors:** 


Scope and Limitations of Cerebral Surgery.—Dr. Kirchhoff has contributed to the *Therapeutische Monatshefte* an article on this subject, a summary of which appears in a recent number of the *Revue Médicale.* He enumerates the varied conditions for which operations on the head have been proposed or carried out, and indicates the reasons for and against operation in each one. Intra-cranial abscess, for example, is a condition in which the advisability of operative interference is not to be disputed, whether the abscess be due to traumatism or result from ear disease, and operation should be carried out as soon as the diagnosis is made. With regard to intra-cranial growths, it is never easy to foretell whether a tumor producing certain symptoms is removable or not. Superficial encapsulated growths are naturally those offering the best opportunities, whilst deeper and more extensive ones do not present the same chance of recovery afterwards, and, on account of the destruction of tissue and the risks from hemorrhage, the operation itself is a formidable one. Epilepsy, when the result of localized lesion of the cortex, is practically the only form of this disease in which a good result is anticipated from operation. Hernia cerebri, Dr. Kirchhoff thinks, may be removed if situated anteriorly, as no paralysis appears to follow. It may also be possible to remove a small one posteriorly. Of course, a condition such as hemorrhage from the middle meningeal artery, if a result of injury, must be treated by trephining, and the same is true of the similar condition associated with pachymeningitis, although it is not easy of diagnosis. The large serous effusions associated with tuberculous meningitis, cerebral tumors, etc., have been evacuated by trephining and puncture, and such an operation may, at least, temporarily relieve the patient. It is doubtful whether trephining for cerebral hemorrhage would be practised even if the difficulty in diagnosticating between that condition and softening could be overcome. Headache, if the pain be localized to some distinct tender point, offers a good opportunity for relief by trephining; but permanent benefit from operation in mental disease has yet to be obtained.—*Medical Age.*

